# Accurate Classification of Multi-Cultivar Watermelons via GAF-Enhanced Feature Fusion Convolutional Neural Networks

**DOI:** 10.3390/foods14162860

**Published:** 2025-08-18

**Authors:** Changqing An, Maozhen Qu, Yiran Zhao, Zihao Wu, Xiaopeng Lv, Yida Yu, Zichao Wei, Xiuqin Rao, Huirong Xu

**Affiliations:** 1 College of Biosystems Engineering and Food Science, Zhejiang University, 866 Yuhangtang Road, Hangzhou 310058, China; 12213004@zju.edu.cn (C.A.); 12313001@zju.edu.cn (M.Q.);; 2 Zhejiang Key Laboratory of Intelligent Sensing and Robotics for Agriculture, Hangzhou 310058, China; 3 Key Laboratory of On-Site Processing Equipment for Agricultural Products, Ministry of Agriculture and Rural Affairs, Hangzhou 310058, China

**Keywords:** multi-cultivar watermelon, Gramian Angular Field, feature fusion, convolutional neural network, wavelength selection

## Abstract

The online rapid classification of multi-cultivar watermelon, including seedless and seeded types, has far-reaching significance for enhancing quality control in the watermelon industry. However, interference in one-dimensional spectra affects the high-accuracy classification of multi-cultivar watermelons with similar appearances. This study proposed an innovative method integrating Gramian Angular Field (GAF), feature fusion, and Squeeze-and-Excitation (SE)-guided convolutional neural networks (CNN) based on VIS-NIR transmittance spectroscopy. First, one-dimensional spectra of 163 seedless and 160 seeded watermelons were converted into two-dimensional Gramian Angular Summation Field (GASF) and Gramian Angular Difference Field (GADF) images. Subsequently, a dual-input CNN architecture was designed to fuse discriminative features from both GASF and GADF images. Feature visualization of high-weight channels of the input images in convolutional layer revealed distinct spectral features between seedless and seeded watermelons. With the fusion of distinguishing feature information, the developed CNN model achieved a classification accuracy of 95.1% on the prediction set, outperforming traditional models based on one-dimensional spectra. Remarkably, wavelength optimization through competitive adaptive reweighted sampling (CARS) reduced GAF image generation time to 55.19% of full-wavelength processing, while improving classification accuracy to 96.3%. A better generalization of the model was demonstrated using 17 seedless and 20 seeded watermelons from other origins, with a classification accuracy of 91.9%. These findings substantiated that GAF-enhanced feature fusion CNN can significantly improve the classification accuracy of multi-cultivar watermelons, casting innovative light on fruit quality based on VIS-NIR transmittance spectroscopy.

## 1. Introduction

Watermelon (*Citrullus lanatus*), rich in water, minerals, and vitamins, is a highly sought-after horticultural crop worldwide [1,2]. Different cultivars of watermelons, including seeded and seedless watermelons, have been widely accepted by consumers. Seeded watermelons are cultivated through natural reproduction and contain black seeds, while seedless watermelons are triploid hybrids with only soft white seeds. Advances in breeding technology have made seedless watermelons increasingly popular for their firm texture, sweetness, and juiciness. Furthermore, seedless watermelon has higher tissue firmness, making it less prone to internal fruit diseases and easier to store or transport, which helps extend its shelf life [3,4,5,6]. In practical sales, there are usually a large number of watermelons on the market that contain different cultivars. Sometimes there is a situation when the two types of watermelons are mixed in transport and sale, or need to be centrally classified according to different needs. Therefore, it is important to explore high-throughput and high-accuracy classification methods of seedless and seeded watermelons.

Seedless and seeded watermelons are very similar in appearance, making it difficult to accurately classify them based on external features like rind color, shape, and size. Modern detection techniques, such as spectral technology [7,8,9], acoustic vibration technology [10,11,12], microwave and millimeter-wave imaging technologies [13], and machine vision technology [14] have been used to study the internal quality of watermelon. Near-infrared spectroscopy can reflect the characteristic information of hydrogen-containing groups (such as C-H, O-H, N-H, S-H, etc.) in organic molecules, thereby reflecting changes in the internal quality of fruit. Additionally, due to the advantages of rapid and non-destructive detection, this technology remains the most widely used method for the online detection of fruit. Owing to the large size and thick rind of watermelon, diffuse reflectance technology has difficulty penetrating the rind to collect information about the flesh, making it challenging to accurately reflect the internal quality of large fruit. This further limits the detection precision of watermelon quality. In transmittance mode, the spectrometer receives light signals that penetrate the whole watermelon. The transmitted light passes through a larger volume of large-sized fruit flesh, carrying more information about its internal quality compared to reflected light [15,16]. What is more, transmittance spectroscopy can achieve high-throughput and online detection, and is more suitable for large-sized fruit. Nowadays, transmittance spectroscopy has been applied to watermelon quality detection, such as for assessing ripeness [8] and sugar content [17]. However, there is a lack of online research using transmittance technology that classifies multi-cultivar watermelons.

In recent years, near-infrared spectroscopy combined with machine learning has been widely used for fruit quality detection [18,19,20,21,22]. Traditional models typically involve multiple complex steps, such as spectral preprocessing and multivariate selection, along with a certain trial-and-error process. This not only consumes a significant amount of time but also requires researchers with specialized knowledge and experience. A key challenge is how to improve the efficiency and versatility of models while increasing detection accuracy. Deep learning has demonstrated a significant information extraction capability across various fields, using deep neural networks to extract high-level features. Tian et al. [19] proposed a one-dimensional convolutional neural network (1D-CNN) and realized the early detection of freezing injury in oranges in combination with VIS/NIR transmission. Similarly, Shi et al. [23] employed near-infrared spectroscopy and developed a CNN model for quality detection across different pear varieties. Guo et al. [24] employed near-infrared spectroscopy and deep learning to enable soluble solid content detection for apple quality assessment at different detection terminals. When using CNN for spectral analysis, most studies focus on one-dimensional CNN for model calibration, largely ignoring the correlation between spectral data features and space.

Recently, researchers have discovered that deep learning offers unique advantages in image processing. With a sufficient number of images, deep learning can capture both local and global features, fusing them from low-dimensional to high-dimensional spaces. This has led to growing interest in converting one-dimensional spectra into two-dimensional images, and combining deep learning for fruit quality detection. Jiang et al. [25] quantitatively analyzed aflatoxin B1 in moldy peanuts based on near-infrared spectroscopy, combing it with a two-dimensional convolutional neural network and using Gramian Angular Summation Field (GASF) to encode the near-infrared spectra. Cai et al. [20] used hyperspectral transmittance imaging and GAF combined with an improved deep learning model to detect early-decayed oranges, achieving an accuracy of 95%.

In this study, it is of great importance to convert 1D spectra to 2D images, so as to enhance cultivar information and remove interference. Specifically speaking, the watermelon characteristics of a large size and thick rind result in weak transmittance spectral signals, and interference variables are more likely to appear in the spectra, making accurate quality detection more challenging. GAF encodes time series data in Cartesian coordinates into polar coordinates, and then calculates the cosine or sine of angles between different points, which will hopefully enhance the signal strength and eliminate interfering signals within the original spectra of the watermelon. Furthermore, the generated GAF images allow for the extraction of key features using CNN. CNN can effectively learn the spatial features and local patterns of images, aiding in more accurate classification of fruit quality [26,27]. Therefore, it is important to explore the image transformation and deep learning method to eliminate interfering factors and improve the accuracy of watermelon classification.

To meet the demand for high-throughput and high-precision classification of multi-cultivar watermelons in practical applications, this study explored classification performance based on transmittance spectroscopy with an improved CNN. This research aimed to (1) analyze the transmittance spectra of seedless and seeded watermelons, (2) explore the classification effect based on the single intensity or absorbance at certain wavelengths and using traditional machine learning methods, and (3) investigate the classification performance by fusing the GAF images and improved CNN, along with a visual analysis of the model.

## 2. Materials and Methods

### 2.1. Watermelon Samples

The ‘*Qilin*’ watermelon, including both seedless and seeded cultivars, was purchased from a local fruit market in Hangzhou, China. Seeded watermelons are diploid plants that undergo fertilization through natural pollination, resulting in the development of mature black seeds. Seedless watermelons are triploid plants cultivated through artificial induction, with only soft white seeds. It should be noted that both seedless and seeded watermelons come from different origins, which is beneficial in improving the general applicability of the model. After being transported to the laboratory, the watermelons were stored for at least 10 h to return to room temperature (25 °C), eliminating any temperature differences that could affect their transmittance spectra.

From July to September 2024, 163 seedless and 160 seeded watermelons were finally obtained for model calibration and prediction in the experiment. With a division ratio of 3:1 using the Kennard–Stone algorithm, the calibration set consists of 122 seedless and 120 seeded watermelons, while the prediction set includes 41 seedless and 40 seeded watermelons. To evaluate the generalization ability of the model, 17 seedless and 20 seeded watermelons from different origins were used for validation. The information of the watermelon samples used for model calibration, prediction, and validation is shown in Table 1.

The mass and diameter of each watermelon sample were measured, followed by the collection of the transmittance spectra. Then, each watermelon sample was cut from the equatorial position to obtain images of the watermelon flesh. As it is difficult to distinguish between seedless and seeded watermelons in appearance, the images were used to further determine the watermelon variety. Finally, the center flesh of the watermelon was obtained, juiced, and tested for its soluble solid content (SSC) using a refractometer (PR-201 α, Atago, Japan). The physical properties and spectra of 163 seedless and 160 seeded watermelons used for model calibration and prediction were subsequently analyzed, excluding the samples used for model validation.

**Figure 1:**
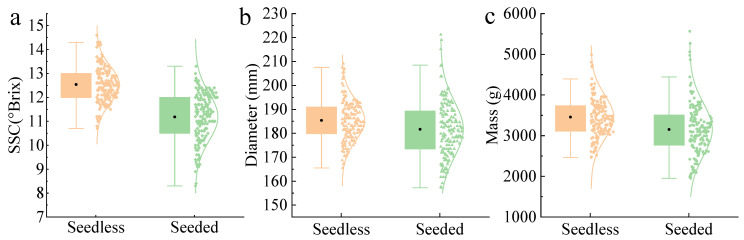
Distribution of SSC (**a**), diameter (**b**), and mass (**c**) of seedless and seeded watermelons.

The statistical results for the SSC, diameter, and mass of the seedless and seeded watermelons are shown in Figure 1a–c, respectively. There were no significant differences in diameter and mass between seedless and seeded watermelons, while considerable variation was observed within samples of the same cultivar. The average SSC of seedless watermelons was higher than that of seeded watermelons, aligning better with the purchasing needs of most consumers.

### 2.2. Transmittance Spectrum Collection

**Figure 2:**
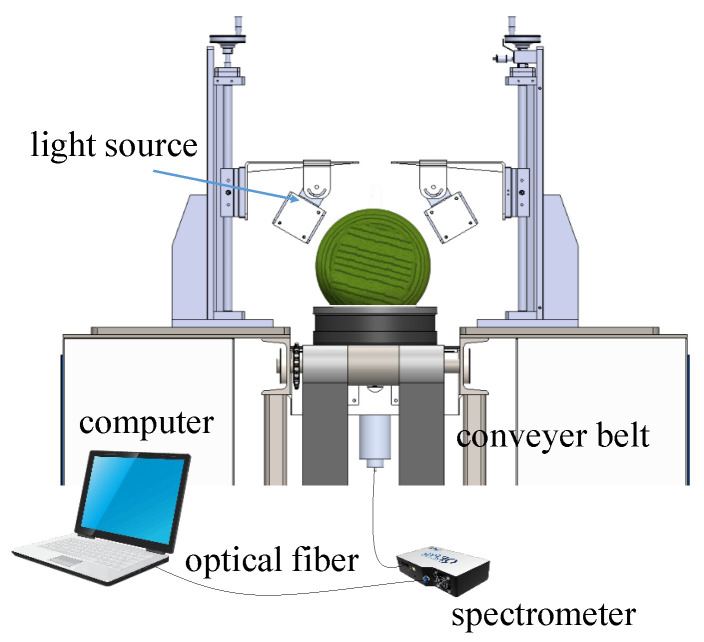
The system for transmittance spectrum collection of watermelon.

The transmittance spectrum collection system is shown in Figure 2. For large-sized fruit with a thick rind, a higher-powered light source is required to obtain a stable and accurate transmittance spectrum. A total of 10 halogen lamps (150 W, 15 V) were symmetrically arranged on both sides of the conveyor belt. No damage to the watermelon caused by the light was observed during the experiment within an integration time of 500 ms. Spectral information was collected and recorded using a spectrometer (QE65PRO, Ocean Optics, Orlando, FL, USA) with a resolution of 0.74 nm through an optical fiber. The transmittance spectrum was collected at 630–1000 nm with 501 wavelength points. Transmittance spectroscopy can capture comprehensive internal information of the watermelon, and in this study, spectra were collected only once for each sample. The raw spectra acquired without pre-processing were used for subsequent model establishment.

In order to eliminate system noise, both the background spectrum and the reference spectrum were taken with a Teflon cylinder. The absorbance spectrum was calculated as shown in Equation (1) [28]:

(1) $$\mathrm{Absorbance}=-\mathrm{lg}\frac{I-I_{B}}{I_{R}-I_{B}}$$

where *I*, *I_B_*, and *I_R_* are the original intensity spectrum of the watermelon, background, and reference, respectively.

### 2.3. Gramian Angular Field

Gramian Angular Field (GAF) is a method used to convert one-dimensional sequences into two-dimensional images, and it is divided into the Gramian Angular Summation Field (GASF) and the Gramian Angular Difference Field (GADF). GAF encodes time series data in Cartesian coordinates into polar coordinates, and then generates a Gramian matrix through trigonometric operation, transforming the data into two-dimensional images. The converted image preserves the time dependence of the data and visualizes certain features of the data. The specific implementation steps of GAF are as follows [20,25].

First, the original transmittance spectrum for each watermelon is obtained. The spectrum is represented as $X=\left\{x_{1},x_{2},x_{3},\ldots ,x_{i},\ldots ,x_{n}\right\}$, where *n* is the number of wavelengths and *x*_*i*_ is the transmittance value. The values are normalized to the range of 0–1 using Equation (2):

(2) $${\tilde{x}}_{i}=\frac{x_{i}-\min(X)}{\max(X)-\min(X)}$$

Then, the parameters of the polar coordinate are calculated by Equation (3), where *α* and *r* are the polar angle and radius, respectively.

(3) $$\left\{\begin{array}{l} \alpha =\arccos({\tilde{x}}_{i})\;0\leq {\tilde{x}}_{i}\leq 1\\ r=\frac{i}{n}\;i\in n \end{array}\right.$$

Two different images can be generated based on different formulas: Gramian Angular Summation Field (GASF) and Gramian Angular Difference Field (GADF), whose expressions are Equations (4) and (5), respectively.

(4) $$GASF=\left[\begin{array}{cccc} \cos(\alpha_{1}+\alpha_{1}) & \cos(\alpha_{1}+\alpha_{2}) & \cdots & \cos(\alpha_{1}+\alpha_{n})\\ \cos(\alpha_{2}+\alpha_{1}) & \cos(\alpha_{2}+\alpha_{2}) & \cdots & \cos(\alpha_{2}+\alpha_{n})\\ \vdots & \vdots & \ddots & \vdots \\ \cos(\alpha_{n}+\alpha_{1}) & \cos(\alpha_{n}+\alpha_{2}) & \cdots & \cos(\alpha_{n}+\alpha_{n}) \end{array}\right]$$

(5) $$GADF=\left[\begin{array}{cccc} \sin(\alpha_{1}-\alpha_{1}) & \sin(\alpha_{1}-\alpha_{2}) & \cdots & \sin(\alpha_{1}-\alpha_{n})\\ \sin(\alpha_{2}-\alpha_{1}) & \sin(\alpha_{2}-\alpha_{2}) & \cdots & \sin(\alpha_{2}-\alpha_{n})\\ \vdots & \vdots & \ddots & \vdots \\ \sin(\alpha_{n}-\alpha_{1}) & \sin(\alpha_{n}-\alpha_{2}) & \cdots & \sin(\alpha_{n}-\alpha_{n}) \end{array}\right]$$

**Figure 3:**
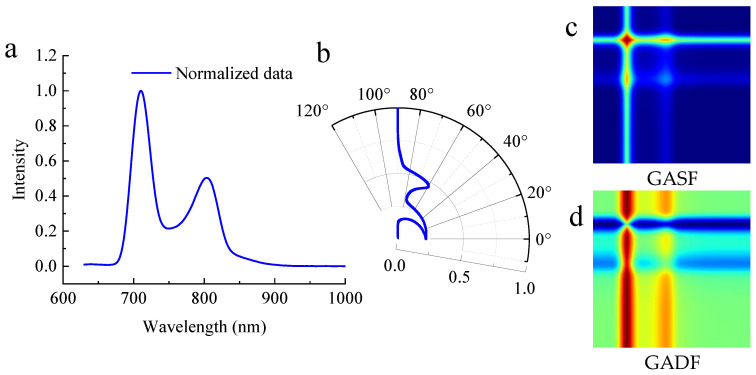
The process of generating a two-dimensional image from a one-dimensional transmittance spectrum. (**a**) Normalized spectrum in the Cartesian coordinate, (**b**) Curve converted to polar coordinate, (**c**) GASF image, (**d**) GADF image.

It can be seen that the encoding position moves from the upper left of the matrix to the lower right corner. To further explain the GAF process, Figure 3 is provided as an example using the transmittance intensity spectrum. The raw normalized spectrum has two distinct intensity peaks (Figure 3a), with the first peak being larger than the second. The two peaks in the polar coordinate of Figure 3b correspond to the spectral peaks, where the first peak in Figure 3a (with an intensity of 1) corresponds to a polar angle of 0°.

In the transformed two-dimensional images (Figure 3c,d), the larger peaks correspond to the lightest and darkest areas, respectively. The generated image corresponds to the matrix values, providing a complete mapping of the one-dimensional spectrum. This mapping includes various features such as points, lines, and colors, which are positioned according to their respective locations in the image.

The spectrum of watermelon is easily interfered by other signals due to its large size. By converting one-dimensional data into two-dimensional images using the Gramian Angular Field, the relationship between angle values corresponding to the spectral intensities in polar coordinates is calculated. This analytical method helps to enhance signal strength while eliminating the effect of interference factors, thereby extracting important features which are beneficial for subsequent data analysis and pattern recognition.

### 2.4. Traditional Model Development

#### 2.4.1. Dimensionality Reduction

Principal component analysis (PCA) is a dimensionality reduction algorithm that transforms multiple variables into a few principal components. These principal components are linear combinations of the original variables and are uncorrelated with each other, while capturing most of the information from the original data [29]. In this study, PCA was used to analyze the original transmittance spectral features of seedless and seeded watermelons.

t-distributed stochastic neighbor embedding (t-SNE) is a dimensionality reduction method used for data visualization and feature extraction. t-SNE measures the similarity between different samples in a high-dimensional space and maps them to a low-dimensional space [30]. t-SNE was used to analyze the spectral characteristics extracted in the CNN of seedless and seeded watermelons.

#### 2.4.2. Wavelength Selection

Competitive adaptive reweighted sampling (CARS) is an efficient algorithm for spectral feature selection [31,32]. The core idea of CARS is to adaptively adjust the selection probability of each spectrum band through Monte Carlo sampling and the exponentially decreasing function (EDF). The optimal band combination that contributes most to modeling performance is ultimately determined based on the minimum RMSECV.

#### 2.4.3. PLS-DA and SVM

Partial least squares discriminant analysis (PLS-DA) is a widely used classification model in machine learning. It employs a classical partial least squares regression model, where the response variable is a set of categorical information that responds to the category relationships between statistical units, and is a supervised discriminant analysis [29].

Support vector machine (SVM), with a radial basis function as the kernel function, is a classical nonlinear classification model. SVM makes data divisible in a high-dimensional feature space by mapping the samples onto that space. Furthermore, the SVM core is for finding an optimal hyperplane in the feature space for classification with maximum separation [33].

### 2.5. Convolutional Neural Network

**Figure 4:**
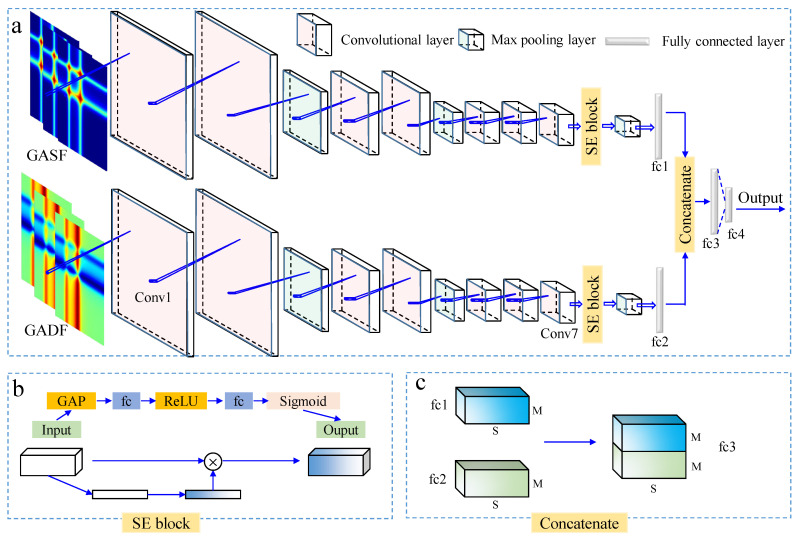
The architecture of the constructed dual-input network model (**a**). The process of SENet (**b**). The process of concatenating two features (**c**). (SE: Squeeze-and-Excitation block, fc: fully connected layer, Conv: Convolutional layer, GAP: global average pooling, M and S are the number of dimensions and channels of feature maps).

CNN, a multilayer parallel neural network, trains the weight parameters of the network through input-to-output mapping relationships, which in turn performs model training. CNN typically consists of convolutional layers, pooling layers, and fully connected layers. The convolutional layer is responsible for extracting related features from the images. The pooling layer compresses the data and reduces the number of parameters, helping to prevent overfitting. The fully connected layer aggregates the low-level features and information obtained from the convolutional and pooling layers. In this study, the 1D-CNN follows the structure described by Tian et al. [19], and the complex 2D-CNN is detailed as follows (shown in Figure 4a).

#### 2.5.1. Single Image Input

The CNN structure is the same for the input of single GASF and GADF images. In this study, the size of the generated GASF and GADF images is 300 × 300 × 3. Referring to the VGG16 [34], a new convolutional neural network model is proposed in this paper. The structure increases the network depth by stacking multiple small-sized convolutional kernels and pooling layers to reduce the spatial dimensions of the images while improving the representation of image features. A relatively small 3 × 3 convolutional kernel and a 2 × 2 maximum pooling kernel are used, with a Rectified Linear Unit (ReLU) function applied after each convolutional layer. At the end of the network, a fully connected layer is designed to map the features extracted by the convolutional and pooling layers to the final classification output. For the classification of seedless and seeded watermelons, in the network with a single image input, the number of nodes in the fully connected layers—fc1 and fc2—is set to 2 (Figure 4a).

The Squeeze-and-Excitation (SE) block, being added behind the last convolutional layer of the CNN, can enhance the network’s focus on important features by adaptively adjusting the weights of the channels, thus improving the model classification performance [35]. As shown in Figure 4b, SE first performs a global average pooling (GAP) on the different channel features of the input. The weight of each channel of the feature layer is then fixed between 0 and 1 by taking a Sigmoid after two fully connected layers (fc). The output layer is finally obtained by multiplying the weight value with the corresponding input layer.

#### 2.5.2. Dual Image Input

To fully extract the feature information from GASF and GADF images, and further improve the classification accuracy of seedless and seeded watermelons, a dual-input CNN model is proposed, as shown in Figure 4a.

Each input (described in Section 2.5.1) independently extracts features from the GASF and GADF images using separate convolutional neural networks. It should be noted that in the dual-input neural network, the number of nodes in the fully connected layers, fc1 and fc2, is set to 100. The features extracted by the two inputs are fused after being processed by the single-input neural network, allowing the combination of features from different GASF and GADF images. As shown in Figure 4c, different feature maps (number of channels is S) are concatenated together according to the channel dimension (number of dimensions is M). At the end of the fused network, two fully connected layers, fc3 and fc4, are designed with 100 and 2 nodes, respectively. The parameters of the constructed dual-input network are shown in Table 2. 

#### 2.5.3. Network Parameter Setting

Parameter setting is critical to model performance. The batch size is set to 10 to reduce memory consumption while maintaining training stability. The number of Epochs is set to 100 to ensure that the model fully learns the data features. The execution environment is selected as GPU to accelerate the training process. A learning rate of 0.001 was used and implemented in the Adam optimizer to optimize parameter updates.

#### 2.5.4. CNN Visualization

Model visualization helps explain the features learned by each convolutional layer, providing deeper insights into the image feature extraction for GASF and GADF images during classification [26]. The first convolutional layer functions similarly to edge detection, where the feature maps in different channels retain most of the image information at this stage. As the layers deepen, the output of the feature maps become more abstract, retaining less information and focusing more on useful features.

### 2.6. Model Evaluation

The same sample division is applied for the comparison of different classification models. The classification performance of different models is evaluated using accuracy, precision, recall, and F1-score. With seedless watermelon defined as Class 1 and seeded watermelon defined as Class 2, these metrics are defined as follows:

(6) $$\mathrm{Accuracy}=\frac{\mathrm{TP}+\mathrm{TN}}{\mathrm{TP}+\mathrm{FP}+\mathrm{TN}+\mathrm{FN}}$$

(7) $$\mathrm{Precision}\text{\_}1=\frac{\mathrm{TP}}{\mathrm{TP}+\mathrm{FP}}\;\mathrm{Precision}\text{\_}2=\frac{\mathrm{TN}}{\mathrm{TN}+\mathrm{FN}}$$

(8) $$\mathrm{Recall}\text{\_}1=\frac{\mathrm{TP}}{\mathrm{TP}+\mathrm{FN}}\;\mathrm{Recall}\text{\_}2=\frac{\mathrm{TN}}{\mathrm{TN}+\mathrm{FP}}$$

(9) $$F1\text{\_}1=2\times \frac{\mathrm{Precision}\text{\_}1\times \mathrm{Recall}\text{\_}1}{\mathrm{Precision}\text{\_}1+\mathrm{Recall}\text{\_}1}F1\text{\_}2=2\times \frac{\mathrm{Precision}\text{\_}2\times \mathrm{Recall}\text{\_}2}{\mathrm{Precision}\text{\_}2+\mathrm{Recall}\text{\_}2}$$

where TP and TN are the number of watermelons correctly classified as seedless and seeded watermelons, respectively. FP is the number of seedless watermelons incorrectly classified as seeded watermelons, and FN is the number of seeded watermelons incorrectly classified as seedless watermelons.

The processes of data analysis and modeling were written based on Python 3.9, and plotting was performed by Origin 2022 (OriginLab Corp., Northampton, MA, USA) on a computer (Intel(R) Xeon(R) Gold 6248 CPU @ 2.50 GHz, NVIDIA RTX A5000 GPU).

## 3. Results and Discussion

### 3.1. Analysis of Spectra

**Figure 5:**
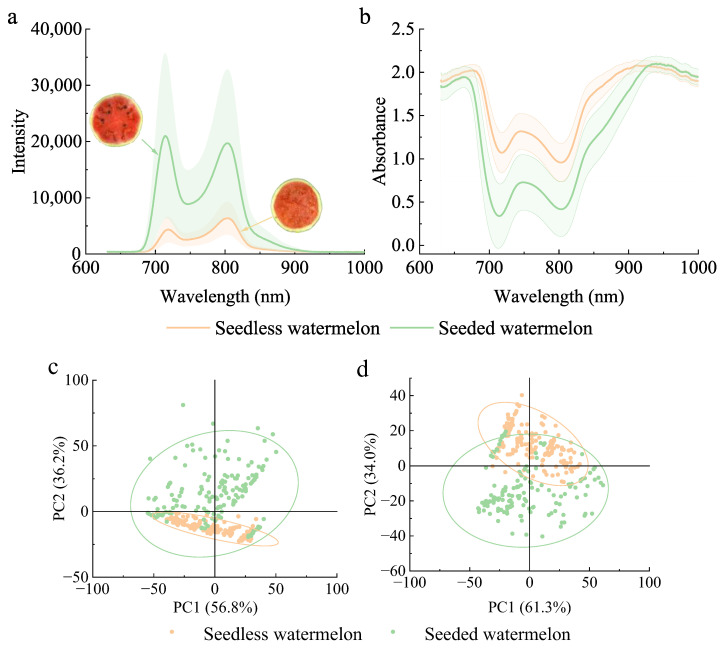
Spectra result of seedless and seeded watermelons: (**a**) Average intensity spectrum, (**b**) Average absorbance spectrum, (**c**) PCA analysis of intensity spectra, (**d**) PCA analysis of absorbance spectra.

Figure 5a and b show the average transmittance intensity and absorbance spectrum of seedless and seeded watermelons in the range of 630–1000 nm, respectively. The data was filled in during the drawing process using Origin software. The transmittance intensity spectra of both watermelons varied similarly with wavelength, exhibiting two intensity peaks around 715 nm and 800 nm. There was an obvious overlap in transmittance intensity between the two types, with the average intensity of seedless watermelon being significantly lower than that of seeded watermelon. As shown in Figure 5b, there was also an overlap in absorbance spectra between different watermelons, with the average absorbance of seedless watermelons being higher than that of seeded watermelons in most wavelength ranges. The absorbance spectra of the watermelons exhibited three distinct absorption peaks around 670 nm, 750 nm, and 920 nm. The absorption peak near 670 nm is associated with chlorophyll, while the peak at 920 nm is ascribed to second overtone O-H stretching from water, and peaks around 760 nm are found to be associated with the overtones of C-H vibrations in carbohydrates [15,28,36]. The spectra differences of the seedless and seeded watermelons benefited the classification of the two types of watermelons.

Differences in textural characteristics between seedless and seeded watermelons resulted in differences in spectral intensity and absorbance. Seedless watermelon has a higher hardness and cell density in both the rind and flesh tissues [4,6], resulting in more absorbed light and a lower transmittance intensity. Additionally, differences in composition between the watermelons, such as sugar content, minerals, and moisture, also have an impact on their transmittance spectra.

Differences in transmittance intensities and absorbance spectra between seedless and seeded watermelons provided the possibility of their classification. Further analyses of the spectral dimensionality reduction using PCA for the watermelons were carried out, as shown in Figure 5c,d. In Figure 5c, PC1 and PC2 explained 93% of the information for all variables. Although the distribution of seedless watermelon was clustered, it completely overlapped within that of the seeded watermelon, making it difficult to distinguish between them, and the result in Figure 5d was the same.

### 3.2. Classification Based on Intensity and Absorbance Value

**Figure 6:**
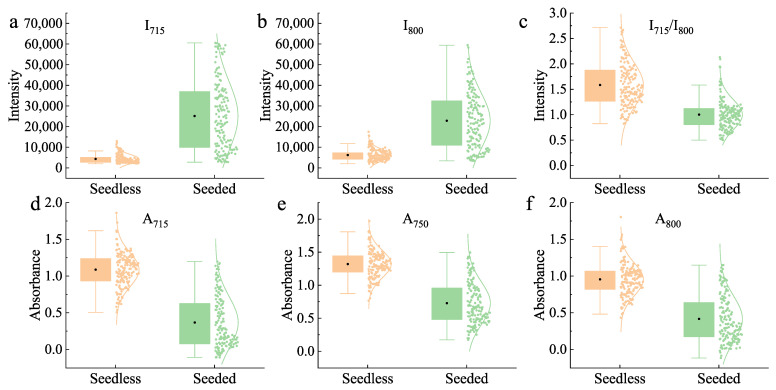
Spectra difference between seedless and seeded watermelon: (**a**) Intensity at 715 nm, (**b**) Intensity at 800 nm, (**c**) Intensity ratio, (**d**) Absorbance at 715 nm, (**e**) Absorbance at 750 nm, (**f**) Absorbance at 800 nm.

The effect of the classification performance of seedless and seeded watermelons based on the spectral intensity and absorbance at a single wavelength is shown in Figure 6. The intensity values at 715 nm and 800 nm were analyzed. The average transmittance intensity of seedless watermelon was significantly lower than that of seeded watermelon. However, there was considerable overlap in the intensity values between the two types, making it difficult to classify them based on the intensity at a single wavelength. The ratio of intensities at the peaks was also used to reflect the internal quality of the watermelons [8]. Figure 6c shows the intensity ratio of seedless and seeded watermelons at 715 nm and 800 nm, indicating that it remained challenging to accurately classify the two types based on the ratio. The average absorbance spectra of the watermelons exhibited significant overlap at 670 nm and 920 nm (Figure 5b). Therefore, absorbance values at the more distinct wavelengths of 715 nm, 750 nm, and 800 nm were selected for analysis, with results presented in Figure 6d–f, respectively. Similarly, the overlapping absorbance values of the seedless and seeded watermelons at different wavelengths made it difficult to make a distinction.

The above result indicated that seedless and seeded watermelons were difficult to classify by intensity or absorbance values, although there was a large difference in average transmittance spectra. It is important to note that the differences in transmittance intensity spectra between seedless and seeded watermelons were significantly larger than those in the absorbance spectra. Furthermore, intensity spectra are more conducive to the classification model [21,37]. Therefore, transmittance intensity spectra were chosen for subsequent modeling.

### 3.3. Classification Result

#### 3.3.1. Models Based on One-Dimensional Spectra

**Figure 7:**
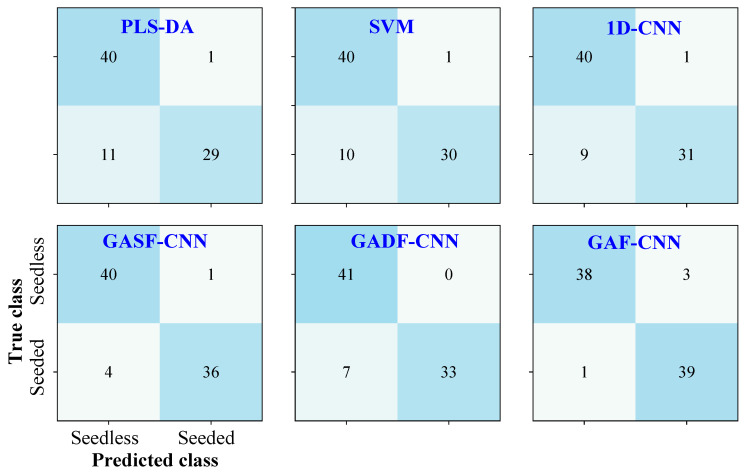
Confusion matrix for the watermelon classification in prediction set using different models.

This study explored the classification results of seedless and seeded watermelons based on one-dimensional spectral data. As shown in Figure 7, PLS-DA and SVM achieved classification accuracies of 85.2% and 86.4% for the prediction set in distinguishing seedless and seeded watermelons, with better performance for classifying seedless watermelons. Compared to PLS-DA and SVM, 1D-CNN provided a slight improvement in classification, with an accuracy of 87.7% in the prediction set. Due to the significant similarity and overlap in the one-dimensional spectral data of seedless and seeded watermelons, the classification performance was unsatisfactory. The models struggled to meet the practical requirements for online classification, resulting in a considerable number of misclassifications between seedless and seeded watermelons.

#### 3.3.2. Models Based on Two-Dimensional Images

The one-dimensional spectral data were transformed into two-dimensional images using the GAF described in Section 2.3. Then, generated GASF and GADF images were used as the single input of the CNN model as described in Section 2.5.1, respectively. As shown in Figure 7, the classification performance of the CNN model for seedless and seeded watermelons was improved using single GASF or GADF images. The GASF-CNN and GADF-CNN model achieved 93.8% and 91.4% classification for the prediction set. Similar to other studies [20,38], the GASF images preserved more attributes of the original data than the GADF images, leading to superior classification performance. The above results indicated that the classification accuracy based on two-dimensional images was higher than that based on one-dimensional spectral data, with both exceeding 90% in the prediction set. This accuracy is sufficient to meet the requirements for the online classification of watermelon. Since the classification accuracy has been improved by using GASF or GADF images as the input of the CNN model alone, it is worth discussing whether the accuracy can be further improved by combining the features of the two images.

As shown in Figure 7, the GAF-CNN achieved a classification accuracy of 95.1% for the prediction set, meeting the requirements for online watermelon classification. The dual-input CNN realized the combination of features from different GASF and GADF images, enhancing the classification performance of the CNN model for seedless and seeded watermelons.

#### 3.3.3. Model Visualization Analysis

**Figure 8:**
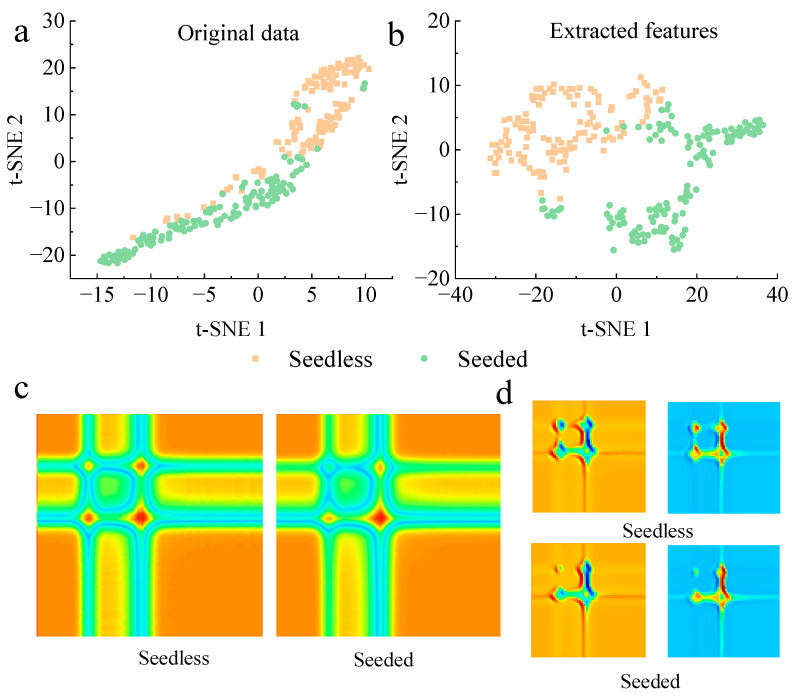
The results of model visualization. Result of t-SNE analysis of original spectral data (**a**) and features extracted in fc3 (**b**). Model visualization of the feature maps of the convolutional layers in Conv1 (**c**) and Conv7 (**d**). The feature maps in Conv7 were selected with the highest weight of each sample after the SE.

Figure 8a,b shows the visualization result of the original transmittance spectra and the features extracted by the fully connected layer (i.e., fc3) of seedless and seeded watermelons in the calibration set, after being mapped into two-dimensional space using t-SNE. While the original spectral data showed a good clustering effect for the watermelons, there was no clear classification boundary, and some samples overlapped and mixed with each other. The features extracted by fc3 showed a clearer distinction between the seedless and seeded watermelons, significantly increasing the separation distance between the different samples.

Using the GASF image as an example, two-dimensional images of a seedless watermelon and a seeded watermelon were randomly selected. As shown in Figure 8c,d, the first convolutional layer of the CNN model-Conv1, and the last convolutional layer-Conv7, displayed one and two feature maps for seedless and seeded watermelons, respectively. Among these, Figure 8d shows the feature maps of the first two channels of Conv7 with the highest weight after the SENet. The first convolutional layer captured the overall feature information of the GASF and GADF images, but without clear distinction. With increases in the convolutional layer depth, the image features captured by the seventh convolutional layer (i.e., Conv7) became increasingly distinct for the two different images. This indicated that the image features learned by the model were refined from global to local, effectively utilizing the two-dimensional image features obtained after GAF transformation.

The visualization analysis demonstrated that after GAF transformation, the constructed CNN model effectively extracted the feature information of seedless and seeded watermelon samples.

#### 3.3.4. CARS-GAF-CNN

**Figure 9:**
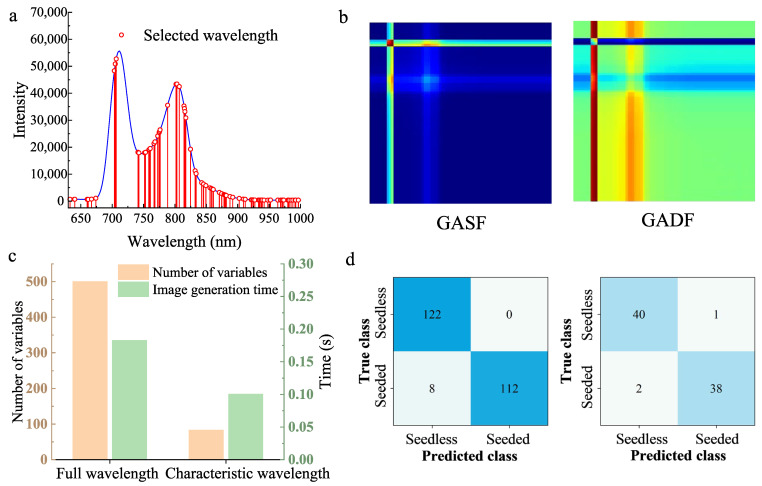
CARS extraction results: (**a**) Selected feature wavelengths, (**b**) Generated GASF and GADF images, (**c**) Differences in the number of variables and image generation time between full wavelengths and characteristic wavelengths using CARS extraction, (**d**) Result of model classification for the calibration and prediction sets.

The original spectrum contained 501 values within the 630–1000 nm range, resulting in a 501 × 501 matrix for the generated GAF image. The large amount of data increased the image generation time and the complexity of the CNN model. In addition, interfering variables in the original data could also affect the classification result. After applying the CARS method, 84 characteristic variables were selected (Figure 9a,c), accounting for only 16.8% of the full spectral data.

The generated GAF images, as shown in Figure 9b, contained less information compared to the images based on the full wavelength range. However, the generated images after the CARS variable extraction displayed more effective and relevant information. The established CARS-GAF-CNN achieved classification accuracies of 96.7% for the calibration set and 96.3% for the prediction set (Figure 9d), with an accuracy improvement of 1.2% compared to the GAF-CNN model.

As shown in Figure 9c, generating a GASF and GADF image for a sample using the original 501 wavelengths took 0.183 s, while using the 84 wavelengths selected by the CARS algorithm reduced the generation time to 0.101 s. The image generation time after optimizing the feature parameters was reduced to 55.19% of the original time. The fewer the feature wavelengths, the more improved the speed of GAF image construction.

### 3.4. Model Comparison

**Figure 10:**
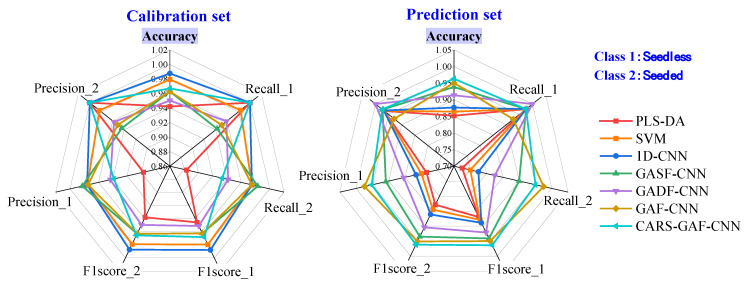
Classification result of the models used in this study for seedless and seeded watermelons in the calibration and prediction sets.

Figure 10 shows the classification result for the calibration and prediction sets of the seven models used in this study. The evaluation metrics were calculated as described in Section 2.6. Models based on GAF images outperformed those based on one-dimensional spectra. After applying CARS variable extraction, a lower generation time of GAF images using fewer wavelengths could be achieved, and the classification accuracy for seedless and seeded watermelons was improved at the same time. Therefore, CARS-GAF-CNN was the optimal model for watermelon classification in this study.

### 3.5. Model Validation Using Different Samples

**Figure 11:**
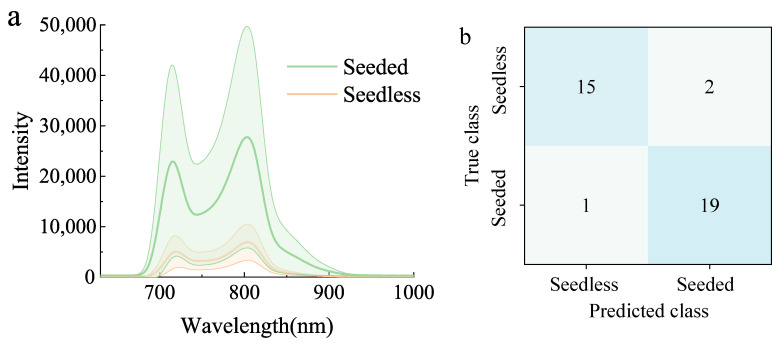
The results of transmittance spectra (**a**) and model classification (**b**) of seedless and seeded watermelons in the validation set.

The independent samples, including 17 seedless and 20 seeded watermelons, were used for model validation. The original transmittance spectra in the validation set are shown in Figure 11a. Although originating from different sources, the watermelons spectra were similar, with a higher average spectrum of seeded watermelons. With spectral data extracted by CARS, the generated GASF and GADF images were used as the input of the established CARS-GAF-CNN model, and the classification results are shown in Figure 11b. The classification accuracy of the CARS-GAF-CNN for the validation set of seedless and seeded watermelons was 91.9%. This satisfactory result shows that the developed model is robust to classify multi-cultivar watermelons of different origins.

### 3.6. Discussion of the Developed CARS-GAF-CNN

#### 3.6.1. Contribution and Interpretability of the Whole Wavelengths

The feature wavelengths extracted by CARS were of higher significance for watermelon classification. The SHapley Additive exPlanations (SHAP) method was introduced to feature interpretability analysis for watermelon classification using 1D-CNN. SHAP quantifies the contribution of intensity features at each wavelength to the model classification, thereby revealing the decision basis of the model and achieving interpretability. The specific process for this can be found in other literature works [39,40].

**Figure 12:**
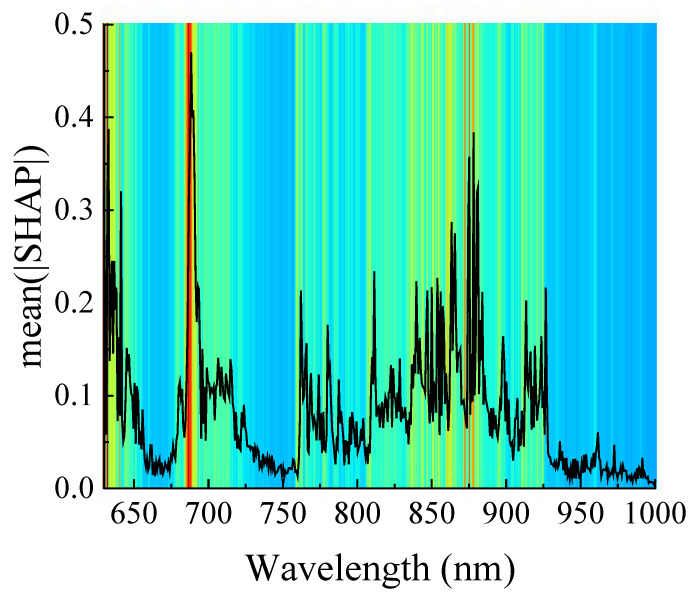
Analysis of the intensity importance at different wavelengths to model classification based on SHAP.

In this study, the global mean absolute SHAP value for watermelon classification was calculated from the full spectra (501 wavelength points) in the prediction set, reflecting the feature importance of each wavelength in the model. The higher the value, the more important it is. As shown in Figure 12, the SHAP values are higher around 630 nm, 675–720 nm, and 800–925 nm, indicating that the intensities in these bands contribute more to watermelon classification. The feature wavelengths extracted by CARS (as shown in Figure 9a) are also mostly included in this range, further demonstrating the effectiveness of the features extracted by CARS.

#### 3.6.2. Advantages of Image Construction

Due to the textural differences between seedless and seeded watermelons, the average transmittance intensity spectra show significant differences (Figure 5), indicating that classification based on transmittance spectra is feasible. However, the one-dimensional spectral signal of watermelon is easily affected by interfering signals. The composition varies significantly at different locations, which in turn affects the reproducibility of spectral signals. Additionally, the signals often carry extensive information such as noise for large-sized watermelons.

Enhancing cultivar variation and removing interfering information from one-dimensional spectral data is of great importance. It has been proven that the model accuracy can be improved by converting one-dimensional spectral data to two-dimensional images through GAF [20,25]. CARS-GAF-CNN is the optimal model used for watermelon classification in this study, with the advantages of (1) Spectral signal enhancement: The one-dimensional spectral signal of watermelon is weak and easily interfered by noise signals, owing to its large size. As introduced in Section 2.3, the applied GAF first calculates the angle value *α* in polar coordinates corresponding to each one-dimensional normalized spectral intensity *x_i_*, where larger intensity values correspond to smaller angle values. The GASF calculates the cosine of angles sum between different points, cos(*α*_*m*_ + *α*_*n*_), enhancing the signal strength through the sum of angles between two points. The GADF computes the sine of the difference between points, sin(*α*_*m*_ − *α*_*n*_). The angle difference between different points helps to eliminate noise and other interfering signals within the original spectra of the watermelon. Therefore, GAF enhances signal information while simultaneously reducing the interference, allowing for the extraction of key features. (2) Multi-input image feature fusion: Compared to one-dimensional spectral data, the generated two-dimensional GAF images are better suited for CNN training and application. Deep learning can more effectively capture spatial information and correlations within the data, thereby improving the accuracy and stability of the model [41,42,43]. Data fusion has been proven to greatly improve the accuracy and robustness of models by combining data with different features [15,44,45,46,47]. GASF images emphasize the overall consistency and positive correlation between time series, while GADF images are suitable for handling negative correlations and change rates between time series, highlighting the dynamic variations in the sequence. By fusing the two-dimensional GASF and GADF images, the model can effectively utilize information from different Gramian Angular Fields, providing a more comprehensive description of the spectra features. This helps extract richer and more distinctive features, thereby improving the classification accuracy between different watermelons.

In summary, important feature wavelengths for watermelon classification were extracted using the CARS, and the GAF further enhanced the information in the one-dimensional spectral data, thereby improving the classification accuracy.

## 4. Conclusions

This study proposed an accurate method for classifying multi-cultivar watermelons with similar appearances. To enhance feature spectral signals and reduce noise interference in one-dimensional spectra, a SE-guided CNN based on GAF encoding and feature fusion was presented. The established GAF-CNN model achieved a classification accuracy of 95.1% for the seedless and seeded watermelons on the prediction set. By utilizing the CARS wavelength optimization, the GAF image generation time was reduced to 55.19% of the full wavelengths, while the classification accuracy was improved to 96.3%. The developed model has good generalization to samples from other origins, with a classification accuracy of 91.9%. The proposed method also provided a new approach for other fruit classification based on transmittance spectra.

Future research will consider more watermelons with different cultivars and growing years to enhance the robustness and reliability of the classification model, so as to achieve the online detection of watermelon quality.

## Figures and Tables

**Table 1:** The information on different watermelon samples.

Sample Set	Cultivar	Number	Origin	Time (Year 2024)
Calibration and prediction	Seedless	163	Jiangsu and Gansu Province	July to September
	Seeded	160	Zhejiang and Hunan Province	July to September
Validation	Seedless	17	Zhejiang Province	October
	Seeded	20	Yunnan Province	December

**Table 2:** The parameters of the constructed dual-input network.

**Layer**	Kernel Size	Stride	Out Channels	Activation	Value
Image					300 × 300 × 3
Conv1, Conv2	3 × 3	1	8	ReLU	
MaxPool1	2 × 2	2			
Conv3, Conv4	3 × 3	1	16	ReLU	
MaxPool2	2 × 2	2			
Conv5, Conv6, Conv7	3 × 3	1	32	ReLU	
MaxPool3	2 × 2	2			
fc1, fc2, fc3			100	ReLU	
drop					0.1
fc4			2	Softmax	

## Data Availability

The original contributions presented in the study are included in the article, further inquiries can be directed to the corresponding author.
